# Association between type of smoking and smoking cessation plans in Korean adults: A nationwide cross-sectional study

**DOI:** 10.18332/tid/199511

**Published:** 2025-02-28

**Authors:** Kitae Park, Dan Bi Kim, Jae Yong Shin, Chung-Mo Nam, Eun-Cheol Park

**Affiliations:** 1 Department of Health Policy and Management, Graduate School of Public Health, Yonsei University, Seoul, Republic of Korea; 2 Department of Public Health, Graduate School, Yonsei University, Seoul, Republic of Korea; 3 Institute of Health Services Research, Yonsei University, Seoul, Republic of Korea; 4 Department of Preventive Medicine, Yonsei University College of Medicine, Seoul, Republic of Korea

**Keywords:** smoking cessation, tobacco, e-cigarettes, dual user

## Abstract

**INTRODUCTION:**

Amid the changing tobacco product landscape, the effects of e-cigarettes on smoking cessation remain unclear. This study aims to examine the relationship between different types of smoking (conventional cigarettes, e-cigarettes, and dual use) and smoking cessation plans among adult smokers.

**METHODS:**

A representative national dataset analysis of KHANES data (2018–2022) was conducted on 1475 current smokers. Current smokers were defined as those who had smoked more than five packs (100 cigarettes) of cigarettes in their lifetime and had smoked in the past 30 days, and type of smoking was classified as conventional cigarette, e-cigarette, or dual use. Smoking cessation plan was classified as ‘yes’ for smokers who intended to quit within one or six months, and ‘no’ for those with no plans to quit. Multivariable logistic regression analysis was used to examine the association between smoking type and smoking cessation plans, adjusting for potential confounders.

**RESULTS:**

A total of 26.0% of male smokers and 30.7% of female smokers had a smoking cessation plan. Compared with conventional cigarette users, e-cigarette users were less likely to have a smoking cessation plan (Male: AOR=0.52; CI: 0.29–0.91; Female: AOR=0.56; CI: 0.16–1.93). The results indicated no statistical significance in female e-cigarette users. In males, e-cigarette users smoking over 20 packs/year were less likely to have smoking cessation plans (AOR=0.11; 95% CI: 0.03–0.58).

**CONCLUSIONS:**

E-cigarette use may have a negative impact on smoking cessation plans in male smokers. The results suggest the importance of public health efforts to provide accurate information and interventions related to e-cigarettes.

## INTRODUCTION

Smoking is the leading cause of premature death and a major preventable one^1,2^. Tobacco contains over 7000 toxic chemicals and generates more than 70 carcinogens, which are inhaled through the respiratory tract. These substances can lead to lung cancer, heart disease, chronic lung disease, and other cancers^3-5^. According to estimates by the World Health Organization (WHO) in 2023, tobacco use is responsible for the deaths of 8 million people each year^6,7^.

To combat the dangers of smoking, WHO member states adopted the WHO Framework Convention on Tobacco Control (WHO FCTC) in 2003^8^. Since signing the WHO Framework Convention on Tobacco Control (FCTC) in 2003 and ratifying it in 2005, South Korea has progressively strengthened tobacco control policies^9^. In Korea, comprehensive tobacco control measures were established by enacting the National Health Promotion Act in 1995, which raised cigarette prices, introduced graphic health warnings on cigarette packages, and provided support for smoking cessation treatments. Subsequently, the country significantly increased the budget for smoking cessation promotion and campaigns.

This has led to increased public interest and demand for reducing the harm caused by tobacco (harm reduction). The tobacco industry claims that e-cigarettes contain 95% less harmful substances than conventional cigarettes, so people should use e-cigarettes. The primary purpose of the tobacco industry’s claim is to protect its interests from various tobacco control policies rather than the health of the people^10^. As one of the means to overcome this crisis, the tobacco industry has developed ‘less harmful’ nicotine-containing products and has been marketing them through various channels, such as articles and social network services, as having reduced health risks^11^. According to the Korea Disease Control and Prevention Agency, the current smoking rate of conventional cigarettes decreased by 1.3% from 37.4% in 2019 to 36.1% in 2023, but the current use rate of electronic cigarettes increased by 4.8% from 9.4% in 2019 to 14.2% in 2023^12^.

However, increasing evidence highlights the potential harms of e-cigarettes. Prior studies have demonstrated adverse health effects of e-cigarettes, including respiratory irritation, increased heart rate, blood pressure, and lung function, which are similar in direction to the effects of traditional smoking^13-15^. This is because the aerosol produced by e-cigarettes contains harmful substances such as formaldehyde, acetaldehyde, and other toxic chemicals that have been linked to respiratory and cardiovascular diseases. Such findings raise critical concerns about the role of e-cigarettes in smoking cessation. While they are often marketed as safer alternatives or cessation tools, their harmful effects may paradoxically discourage smokers from pursuing or sustaining smoking cessation efforts. Moreover, the addictive properties of nicotine in e-cigarettes could undermine the motivation to quit entirely, creating a dependence on e-cigarettes instead^16^. Therefore, it is essential to examine how e-cigarette use influences smoking cessation plans, particularly in the context of their dual role as both perceived aids and potential barriers to quitting.

This study aimed to systematically analyze the association between different types of smoking (cigarettes, e-cigarettes, dual use) and smoking cessation plans using representative data from the Korean population. Although e-cigarettes have demonstrated some potential as a smoking cessation tool, the effects of complex factors such as smoking quantity and dual use on smoking cessation behavior and plans, have not been identified. Therefore, this study aimed to evaluate the effects of e-cigarettes and dual use on smoking cessation plans compared to cigarette users.

## METHODS

### Study population

A secondary dataset analysis was conducted using the Korea National Health and Nutrition Examination Survey (KNHANES), a nationwide cross-sectional survey conducted annually by the Korea Centers for Disease Control and Prevention (KCDC)^17^. The survey targets a new sample of approximately 10000 individuals aged 1 year and older. The sampling strategy follows a probability design that is both multi-stage and clustered. KHANES data examined specific lifestyle factors, such as smoking, alcohol consumption, physical activity, and dietary habits^17^. The ethics approval for the KNHANES was waived by the KCDC Institutional Review Board under the Bioethics and Safety Act and opened to the public in 2018. Our analysis focused on the KNHANES (2018–2022), which provides the most recent data on smoking patterns.

Of the 11162 adults aged 19 years and older who participated in KNHANES (2018–2022), data on never-smoking adults (n=9613) were excluded from our analysis. We excluded adults who did not respond to the survey (n=74). Therefore, our study included 1475 adults (males: 1214, females: 261).

### Type of smoking

Participants were current smokers. The survey asked participants whether they had smoked more than 100 cigarettes (5 packs) in their lifetime, and those who answered ‘yes’ were defined as current smokers^18^. In our study, the independent variable was the type of smoking, which was categorized into three groups: conventional cigarette (CC), electronic cigarette (EC), and dual use. Participants who answered ‘yes’ to either ‘Have you ever smoked regular cigarettes?’ or ‘Have you ever inhaled electronic cigarettes?’, were asked the follow-up question, ‘Have you smoked regular/electronic cigarettes in the past 30 days?’. Participants were classified as ‘CC users’ or ‘EC users’. Those who answered ‘yes’ to both were also classified as ‘Dual users’.

### Smoking cessation plan

The dependent variable was the smoking cessation plan. We categorized smoking cessation plans based on the Transtheoretical Model^19^. The stages of this model are pre-consideration (no plan to quit smoking within 6 months), consideration (planning to quit smoking within 6 months), preparation (planning to quit smoking within 1 month), action (continuation period after quit attempt is less than 6 months), and maintenance (continuation period of quitting smoking is 6 months or more)^20^. We classified participants who answered, ‘I plan to quit smoking within one month’ or ‘I plan to quit smoking within six months’ as ‘planning to quit smoking’ and those who answered, ‘I plan to quit smoking someday, but not within six months’ or ‘I have no plans to quit smoking’, as ‘not planning to quit smoking’.

### Covariates

We considered the following sociodemographic characteristics: sex (male, female), age (19–29, 30–39, 40–49, ≥50 years), marital status (living with spouse, single, or widow), education level (high school or lower, college or higher), household income (low, middle low, middle high, high), residence (urban, rural), and occupational categories (white, pink, blue, unemployed), and survey year (2018, 2019, 2020, 2021, 2022). Health-related behaviors were assessed as physical activity (yes, no), body mass index (BMI) (normal, underweight, obese), alcohol intake (yes, no), age started to smoke (<19, ≥19 years), and stress (yes, no).

### Statistical analysis

We compared the differences in general characteristics according to the smoking cessation plan using χ^2^ tests. We analyzed the association between smoking type and smoking cessation plans using multivariable logistic regression to estimate adjusted odds ratios (AORs) and 95% CIs. We stratified by socioeconomic status (education level, household income, and occupational category) and used multivariable logistic regression models to examine changes in associations. Multivariable models were adjusted for potential confounders, including age, marital status, education level, household income, residence, occupational categories, physical activity, BMI, alcohol intake, age started smoking, stress levels, and survey year. Subgroup analyses were also performed, stratified by packs/year. We performed multinomial logistic regression, categorizing the duration of smoking cessation into three (no: >6 months; contemplation: 1–6 months; preparation: <1 month). All analyses were performed using SAS version 9.4 (SAS Institute, Inc., Cary, NC, USA), and all statistical tests were 2-sided with a significance threshold of p<0.05.

## RESULTS

Table 1 presents the general characteristics of the study population, stratified by sex, using χ^2^ tests. Of the 1475 participants, 316 (26.0%) male smokers and 80 (30.7%) female smokers reported having a smoking cessation plan. Among male smokers, 739 (60.9%) were CC users, 147 (12.1%) were EC users, and 328 (27.0%) were dual users. Similarly, among female smokers, 161 (61.7%) were CC users, 24 (9.2%) were EC users, and 76 (29.1%) were dual users (Table 1).

**Table 1:** General characteristics of the study population in 2018–2022 KNHANES (N=1475)

*Characteristics*	*Smoking cessation plan*													
	*Male*							*Female*						
	*Total*		*No*		*Yes*		*p*	*Total*		*No*		*Yes*		*p*
	*n*	*%*	*n*	*%*	*n*	*%*		*n*	*%*	*n*	*%*	*n*	*%*	
	*1214*	*100*	*898*	*74.0*	*316*	*26.0*		*261*	*100*	*181*	*69.3*	*80*	*30.7*	
**Type of smoking**							0.0122							0.1041
Conventional cigarettes	739	60.9	542	73.3	197	26.7		161	61.7	104	64.6	57	35.4	
E-cigarettes	147	12.1	123	83.7	24	16.3		24	9.2	18	75.0	6	25.0	
Dual use	328	27.0	233	71.0	95	29.0		76	29.1	59	77.6	17	22.4	
**Age** (years)							0.2946							0.2157
19–29	370	6.4	270	73.0	100	27.0		123	47.1	80	65.0	43	35.0	
30–39	341	5.9	265	77.7	76	22.3		79	30.3	59	74.7	20	25.3	
40–49	269	4.6	192	71.4	77	28.6		40	15.3	26	65.0	14	35.0	
≥50	234	4.0	171	73.1	63	26.9		19	7.3	16	84.2	3	15.8	
**Marital status**							0.2401							0.7967
Living with spouse	609	10.5	441	72.4	168	27.6		96	36.8	68	70.8	28	29.2	
Single/widow	605	10.4	457	75.5	148	24.5		165	63.2	113	68.5	52	31.5	
**Education level**							0.4283							0.5295
High school or lower	636	1063	477	75.0	159	25.0		172	65.9	122	70.9	50	29.1	
College or higher	578	847	421	72.8	157	27.2		89	34.1	59	66.3	30	33.7	
**Household income**							0.7244							0.5716
Low	98	1.7	74	75.5	24	24.5		35	13.4	25	71.4	10	28.6	
Middle low	280	4.8	200	71.4	80	28.6		84	32.2	61	72.6	23	27.4	
Middle high	377	6.5	283	75.1	94	24.9		82	31.4	52	63.4	30	36.6	
High	459	7.9	341	74.3	118	25.7		60	23.0	43	71.7	17	28.3	
**Residence**							1.0000							0.1759
Urban	580	10.0	429	74.0	151	26.0		132	50.6	86	65.2	46	34.8	
Rural	634	10.9	469	74.0	165	26.0		129	49.4	95	73.6	34	26.4	
**Occupational categories**							0.0802							0.8022
White-collar	420	7.2	297	70.7	123	29.3		79	30.3	55	69.6	24	30.4	
Pink-collar	222	3.8	175	78.8	47	21.2		76	29.1	55	72.4	21	27.6	
Blue-collar	347	6.0	265	76.4	82	23.6		25	9.6	18	72.0	7	28.0	
Unemployed	225	3.9	161	71.6	64	28.4		81	31.0	53	65.4	28	34.6	
**Physical activity**							0.7105							0.2263
Yes	418	7.2	306	73.2	112	26.8		49	18.8	38	77.6	11	22.4	
No	796	13.7	592	74.4	204	25.6		212	81.2	143	67.5	69	32.5	
**Body mass index**							0.8539							0.2275
Normal	608	10.5	454	74.7	154	25.3		173	66.3	116	67.1	57	32.9	
Underweight	33	0.6	24	72.7	9	27.3		25	9.6	21	84.0	4	16.0	
Obese	573	9.9	420	73.3	153	26.7		63	24.1	44	69.8	19	30.2	
**Alcohol intake**							0.8111							1.0000
Yes	976	16.8	720	73.8	256	26.2		197	75.5	137	69.5	60	30.5	
No	238	4.1	178	74.8	60	25.2		64	24.5	44	68.8	20	31.3	
**Age started smoking**							0.1421							0.3804
<19	671	11.6	508	75.7	163	24.3		133	51.0	96	72.2	37	27.8	
≥19	543	9.4	390	71.8	153	28.2		128	49.0	85	66.4	43	33.6	
**Stress**							0.8020							0.1004
Yes	429	7.4	315	73.4	114	26.6		139	53.3	103	74.1	36	25.9	
No	785	13.5	583	74.3	202	25.7		122	46.7	78	63.9	44	36.1	
**Year of survey**							0.1148							0.5243
2018	311	5.4	223	71.7	88	28.3		67	25.7	44	65.7	23	34.3	
2019	285	4.9	199	69.8	86	30.2		49	18.8	35	71.4	14	28.6	
2020	233	4.0	174	74.7	59	25.3		55	21.1	38	69.1	17	30.9	
2021	209	3.6	164	78.5	45	21.5		50	19.2	37	74.0	13	26.0	
2022	176	3.0	138	78.4	38	21.6		40	15.3	27	67.5	13	32.5	

Table 2 presents the results of the multivariate logistic regression analysis conducted to examine the association between the type of smoking and smoking cessation plan. EC user was positively associated lower likelihood of reporting smoking cessation plans compared with CC users (Male: AOR=0.52; 95% CI: 0.29–0.91; Female: AOR=0.56; 95% CI: 0.16–1.93).

**Table 2:** Results of multiple logistic regression of smoking status and smoking cessation plan by gender, among smokers in 2018–2022 KNHANES (N=1475)

*Variables*	*Male*	*Female*
	*AOR (95% CI)*	*AOR (95% CI)*
**Conventional cigarette user** (ref.)	1	1
**E-cigarette user**	0.52 (0.29–0.91)	0.56 (0.16–1.93)
**Dual user**	1.02 (0.73–1.41)	0.47 (0.22–1.01)

AOR: adjusted odds ratio; adjusted for age, marital status, education level, household income, residence, occupational categories, physical activity, BMI, alcohol intake, age started smoking, stress levels, and survey year.

Table 3 shows the results of subgroup analyses stratified by socioeconomic status for smoking cessation plans. Among male, EC users with an education level of college or higher (AOR=0.36; 95% CI: 0.17–0.78), middle-low household income (AOR=0.13; 95% CI: 0.02–0.67), and blue-collar occupation (AOR=0.16; 95% CI: 0.03–0.72) were less likely to plan to quit compared with a CC user.

**Table 3:** Results of smoking status and smoking cessation plan by gender, stratified by socioeconomic status, in 2018–2022 KNHANES (N=1475)

*Variables*	*Male*					*Female*				
	*CC user*	*EC user*		*Dual user*		*CC user*	*EC user*		*Dual user*	
		*AOR*	*95% CI*	*AOR*	*95% CI*		*AOR*	*95% CI*	*AOR*	*95% CI*
**Education level**										
High school or lower	1	0.85	0.35–2.04	1.30	0.80–2.11	1	0.43	0.08–2.29	0.41	0.16–1.07
College or higher	1	0.36	0.17–0.78	0.91	0.57–1.45	1	0.45	0.05–3.95	0.48	0.11–2.01
**Household income**										
Low	1	0.44	0.02–8.86	0.96	0.29–3.12	1				
Middle low	1	0.13	0.02–0.67	1.39	0.71–2.71	1	0.17	0.01–3.94	1.96	0.37–10.24
Middle high	1	0.58	0.23–1.43	0.79	0.43–1.46	1			0.11	0.02–0.46
High	1	0.52	0.21–1.33	0.91	0.52–1.58	1				
**Occupational categories**										
White-collar	1	0.47	0.22–1.02	0.90	0.51–1.57	1			0.44	0.07–2.59
Pink-collar	1	0.15	0.01–1.30	1.05	0.46–2.41	1				
Blue-collar	1	0.16	0.03–0.72	1.31	0.69–2.48	1				
Unemployed	1	1.29	0.35–4.73	0.91	0.41–2.00	1			0.50	0.10–2.41

CC: conventional cigarette. EC: e-cigarette. AOR: adjusted odds ratio; adjusted for age, marital status, education level, household income, residence, occupational categories, physical activity, BMI, alcohol intake, age started smoking, stress levels, and survey year.

To further examine the impact of smoking history in packs/year on smoking cessation plans, we performed subgroup analyses. Overall, a smoking history of ≥20 packs/year was associated with a lower likelihood of planning to quit in male smokers (EC users: AOR=0.11; 95% CI: 0.03–0.58; Dual users: AOR=0.63; 95% CI: 0.35–1.14) (Table 4).

**Table 4:** Association between smoking status stratified by packs/year and smoking cessation plan by gender, in 2018–2022 KNHANES (N=1475)

*Variables*	*Male*			*Female*		
	*n*	*AOR*	*95% CI*	*n*	*AOR*	*95% CI*
**CC user** (ref.)	739	1		161	1	
**EC user** (packs/year)						
<20	114	0.65	0.36–1.20	24	0.56	0.18–1.79
≥20	33	0.11	0.03–0.58	0		
**Dual user** (packs/year)						
<20	245	1.18	0.81–1.73	74	0.47	0.21–1.11
≥20	83	0.63	0.35–1.14	2		

CC: conventional cigarette. EC: e-cigarette. AOR: adjusted odds ratio; adjusted for age, marital status, education level, household income, residence, occupational categories, physical activity, BMI, alcohol intake, age started smoking, stress levels, and survey year.

## DISCUSSION

E-cigarettes can cause potential harm, such as encouraging the continued use of tobacco products and normalizing smoking behavior. In this cross-sectional study of a nationally representative sample of Korean adults, we found that e-cigarette use in male smokers was associated with a lower likelihood of reporting smoking cessation plans. This finding may reflect the influence of both past and current smoking behaviors, suggesting that e-cigarettes may undermine the intention to quit smoking. These results align with previous studies indicating that e-cigarette use could serve as a barrier to smoking cessation, potentially due to the addictive nature of nicotine and the reinforcement of smoking habits^21^.

Our study demonstrated some differences compared with previous studies on the effects of e-cigarette use on plans to quit. First, we did not find an association between dual smoking and smoking cessation plans. Previous studies asserted that dual smokers are associated with attempts to quit smoking over the past year but not with smoking cessation rates over the past 30 days^22^. These discrepancies may be due to differences in the definition of current smokers and smoking cessation (smoking cessation plans vs quit attempts) or variations in the study design. Specifically, previous studies were conducted on American populations and defined dual users as those who use both combustible cigarettes and smokeless tobacco products (e.g. moist snuff, dip, spit, chew tobacco or snus). In contrast, our study defines dual users as individuals who use both combustible cigarettes and electronic cigarettes. Second, we found that being male was statistically significantly associated with decreased e-cigarette use and smoking cessation plans, whereas no such association was observed for females. This result may be due to the low number of female smokers. We found a large difference in smoking between the sexes, with 82.3% of the 1475 current smokers being male and only 17.7% being female. Korea has been reported to have the highest male smoking rate and lowest female smoking rate among member countries of the Organization for Economic Co-operation and Development, which may be due to the negative perception of female smoking in Korea. Thus, the number of female smokers may be underestimated; additional research is needed to accurately report on female smoking^23,24^.

In our subgroup analysis stratified by socioeconomic status, we found that college or higher level of education, low household income, and employment were associated with a lower likelihood of reporting smoking cessation plans among male e-cigarette users compared to male conventional cigarette smokers. These findings partially contradict the smoking harm paradox that smokers from low socioeconomic backgrounds experience disproportionate health damage^25^. These discrepancies may be explained by complex interactions between socioeconomic factors and smoking behavior. For example, smokers with higher level of education may not perceive e-cigarettes as less harmful than cigarettes, and thus may not embrace e-cigarettes as a means of quitting^26^. On the other hand, among smokers with lower household income, e-cigarette use may have a higher initial cost than cigarettes, which may reduce motivation to quit if the immediate economic and smoking cessation benefits are unclear^27^. Due to their work environments, employees may find e-cigarettes a more practical option than conventional cigarettes. E-cigarettes are often chosen as a convenient alternative for continuing to smoke, especially in places where smoking is restricted. This makes e-cigarettes a preferred choice for smokers looking to maintain their habit without violating workplace policies rather than serving as a tool for quitting.

Although prior research identified a statistically significant difference in nicotine dependence based on the time to first cigarette (TTFC), it did not clarify which type of smoking exhibited the highest nicotine dependence rate, as no clear trend toward an increase or decrease in TTFC was observed. In contrast, our study stratified the participants by packs/year and examined the trend in nicotine dependence across different strata, offering a more detailed analysis of the association^28^. Our study found that male e-cigarette users with a past smoking history of ≥20 packs/year were less likely to report plans to quit smoking. This highlights the importance of considering smoking history in cessation interventions for e-cigarette users. This finding is similar to that of a previous study reporting that prolonged use of e-cigarettes is associated with smoking cessation difficulties^29^.

The possible explanations for such difficulties include, first, a higher nicotine dependence in e-cigarette users with a smoking history of ≥20 packs/year or more. These smokers may have attempted to quit smoking multiple times in the past but failed owing to nicotine addiction, which could make smoking cessation plans more challenging. Second, smokers may be influenced by favorable attitudes toward e-cigarettes and low barriers to e-cigarette smoking in private and smoke-free public settings^30^. E-cigarette users may gain greater social acceptance from nonsmokers who are averse to the smell of conventional cigarettes, allowing them to use e-cigarettes more freely in public places. For instance, in the US, more than 60% of e-cigarette users, and approximately 27% in the United Kingdom and Australia, have reported smoking in smoke-free public spaces^31,32^. However, the amount of nicotine inhaled from e-cigarettes is variable and unpredictable, necessitating a regulatory system that takes into account the nicotine content and ingredients of e-cigarettes^26^. Owing to data limitations, we were unable to investigate the nicotine content of e-cigarettes, but prior research has suggested that high-nicotine e-cigarette use may actually stimulate interest in cigarettes^33^.

### Strengths and limitations

This study has methodological limitations that should be considered when interpreting the results. First, given the cross-sectional design of the study, we could not clarify the association between e-cigarette use and smoking cessation. Additional longitudinal studies are needed to verify this aspect. Second, self-reports of smoking history in the female sample may have been underestimated owing to social desirability response bias. Similarly, while the sample reflects the South Korean population, the findings may not be fully generalizable to other countries or settings due to differences in smoking behaviors, health policies, and sociocultural factors. Third, despite adjusting for various potential confounders in the multivariable models, residual confounding may still exist due to unmeasured or inadequately measured factors.

Nevertheless, this study has several strengths. First, the use of nationally representative data increases the generalizability to male smokers. Second, the analysis of multiple subgroups by socioeconomic status, smoking history, and duration of smoking cessation may help in the formulation of specific and diverse tobacco interventions.

## CONCLUSIONS

This cross-sectional study found that e-cigarettes were associated with lower smoking cessation intentions compared to conventional cigarettes. Our findings highlight the importance of continued public health interventions regarding the harmful effects of tobacco products.

## Data Availability

The data supporting this research are available from the following source: https://knhanes.kdca.go.kr/knhanes/main.do.
